# Use of Proton Pump Inhibitors Is Not Associated with Post-Dialysis Fatigue and Time of Recovery after Dialysis in Patients on Maintenance Hemodialysis

**DOI:** 10.3390/jcm13113241

**Published:** 2024-05-30

**Authors:** Maurizio Bossola, Ilaria Mariani, Tania Monteburini, Emanuele Parodi, Stefano Santarelli, Vittorio Sirolli, Stefano Cenerelli, Mario Bonomini, Silvia Tedesco, Claudia Spoliti, Enrico Di Stasio

**Affiliations:** 1 Servizio Emodialisi, Divisione di Nefrologia, Università Cattolica del Sacro Cuore, 00168 Rome, Italy; 2 Fondazione Policlinico Universitario A. Gemelli IRCCS, 00168 Rome, Italy; claudia.spoliti@policlinicogemelli.it (C.S.);; 3 Dipartimento di Nefrologia, Ospedale “Carlo Urbani”, 60035 Jesi, Italy; 4 Dipartimento di Nefrologia, Ospedale Civile, 15121 Alessandria, Italy; 5 Dipartimento di Nefrologia, Università di Chieti, 66100 Chieti, Italy; 6 Dipartimento di Nefrologia, Ospedale “Civile”, 60019 Senigallia, Italy; 7 Divisione di Chimica, Biochimica, e Biochimica Molecolare, Università Cattolica del Sacro Cuore, 00168 Rome, Italy

**Keywords:** post-dialysis fatigue, proton pump inhibitors, time of recovery, hemodialysis

## Abstract

**Objectives**: To define if the use of proton pump inhibitors (PPI) is associated with PDF prevalence and characteristics and with time of recovery after dialysis in patients on maintenance hemodialysis. **Methods**: Patients were defined as experiencing PDF if they spontaneously offered this complaint when asked the open-ended question: “Do you feel fatigued after dialysis?”. Time of recovery after dialysis (TIRD) was also assessed for each patient. Each patient was invited to rate the intensity, duration and frequency of PDF from 1 to 5. We defined if patients used PPI (no PPI use or PPI use), the type of used PPI, the dose of used PPI, and the duration of the use of PPI (<1 year or ≥1 year). **Results**: A total of 346 patients were studied: 259 used PPI (55 used omeprazole, 63 esomeprazole, 54 pantoprazole, 87 lansoprazole, and 7 rabeprazole) and 87 did not. Two hundred and thirty-two patients declared PDF and 114 did not. The median [min–max] TIRD was 210 min [0–1440]. The prevalence of PDF in PPI users and PPI non-users was 67% and 68%, respectively (*p* = 0.878). The median [min–max] TIRD did not differ significantly between PPI users and PPI non-users (180 [0–1440] and 240 [0–1440], respectively; *p* = 0.871). Median PDF intensity, duration, frequency, and severity did not differ significantly between PPI use and no use. The prevalence of PDF was similar among the different types of PPI use and did not differ with respect to PPI non-users. Duration of PPI exposure was <1 year in 40 patients and ≥1 year in 219 patients. The prevalence of PDF did not differ between the two exposures. The correlation matrix between PPI equivalent dose, PPI treatment duration and PDF frequency, PDF characteristics, and TIRD showed whether there was statistical significance. **Conclusions**: The use of PPI is not associated with PDF and time of recovery after dialysis in patients on maintenance hemodialysis.

## 1. Introduction

Following a hemodialysis treatment, many patients report feeling tired and needing rest or sleep. This condition, conventionally named post-dialysis fatigue (PDF), has been described as a feeling of being worn out, drained or exhausted [1,2,3]. PDF is one of the most debilitating symptoms of hemodialysis patients, significantly impairing their quality of life and causing frustration and depression [1,2,3].

The causes and the pathogenesis of PDF are essentially unknown [1,2,3,4]. However, some mechanisms have been proposed, such as the rapid decline in osmolarity occurring during dialysis as result of the combined effect of a reduction in serum urea and sodium concentration (this leads to brain swelling and headache, restlessness, nausea, muscle cramps and fatigue [5,6]; the release of cytokines such as interleukin-1, interleukin-6 and tumor necrosis factor-alpha [7,8,9]; the accumulation of metabolites and toxins in the muscle [10]).

The characteristics of PDF, intensity, duration and frequency, are elevated in a large percentage of patients, suggesting that PDF is an intense event in terms of quantity and quality [11]. In addition, PDF is independently associated with the time to recover after dialysis (TIRD) [11]. Although PDF and TIRD are not the same, TIRD may be used easily as an indirect measure of PDF.

Recently, it has been demonstrated that the use of proton pump inhibitors (PPI) is independently associated with fatigue severity, a more than 2-fold higher risk of severe fatigue, and lower physical and mental health-related quality of life among kidney transplant recipients, independent of potential confounders [12].

PPI are widely used also in end-stage renal disease (ESRD) patients on maintenance hemodialysis, worldwide [13]. Thus, we conducted the present study with the aim to define if the use of PPI in patients on maintenance hemodialysis is associated with PDF prevalence and characteristics and with the time of recovery after dialysis.

## 2. Patients and Methods

All prevalent ESRD patients receiving maintenance hemodialysis at our five hospitals on December 2023 were considered eligible for inclusion in this study. Exclusion criteria were as follows: dialysis duration <1 year, diagnosis of dementia based on DSM-IV criteria, presence of acute infectious disease, presence of active cancer, vascular access though a central venous catheter, heart failure (class 3 and 4 heart failure according to New York Heart Association classification), respiratory failure, hemorrhage, severe infections, alcohol abuse, shock and liver disease (compensated or decompensated cirrhosis). This study was performed in adherence to the Declaration of Helsinki and the protocol was approved by the local ethics committee. Written informed consent was obtained from all participants before enrollment in this study. For each participant, the following parameters were recorded at the time of inclusion in this study: age, dialytic age, gender, underlying renal disease, weight, height, interdialytic weight gain, body mass index, type and number of comorbid conditions, the Charlson comorbidity index [14], the functional ability, and the symptoms of depression.

### 2.1. Hemodialysis

All patients were receiving conventional 4 h bicarbonate hemodialysis, three times a week. The blood flow ranged from 250 to 300 mL/min with a dialysis rate flow of 500 mL/min. All patients were treated with high-permeability membranes. Membranes were not reused.

### 2.2. PPI Use

For all patients, we defined if they used PPI (no PPI use or PPI use), the type of used PPI (omeprazole, esomeprazole, pantoprazole, lansoprazole, and rabeprazole), the dose of used PPI, and the duration of the use of PPI (<1 year or ≥1 year). In accordance with the study of Knobbe et al., daily PPI dose was calculated in omeprazole equivalents as follows: 1.00 for omeprazole (reference), 0.23 for pantoprazole, 1.60 for esomeprazole, 1.82 for rabeprazole, and 0.90 for lansoprazole [12].

### 2.3. Identification and Grading of PDF

The assessment of PDF was conducted according to the studies of Sklar et al. [15]. Each patient was interviewed during one of the patient’s regularly scheduled treatment. Patients were defined as experiencing PDF if they spontaneously offered this complaint when asked the open-ended question: “Do you feel fatigued after dialysis?”. Then, each patient was invited to rate the intensity, duration and frequency of PDF from 1 to 5. Then, we calculated the sum of scores of PDF intensity, duration and frequency and defined it as PDF severity. The recovery time after the hemodialysis session (TIRD) was calculated according to Lindsay et al. [16]. Briefly, patients were invited to answer to the following single open-ended question: “How long does it take you to recover from a dialysis session?”. Responses were subsequently converted into the number of minutes [16].

### 2.4. Other Measurements

Functional ability was estimated using the Katz’ activities of daily living (ADLs), and the Lawton and Brody scale for instrumental activities of daily living (IADLs) [17,18]. These scales are most commonly adopted for assessing functional independency for clinical and epidemiological purposes; disability in the ADLs was defined as the need for assistance to perform two or more ADLs. The reason for not choosing a single-point decline is that impairment in two ADLs is less likely to capture physiological fluctuations in functional performance. Impairment in IADL function was identified by a score < 7; this higher cut-off level is generally adopted to avoid a “floor effect”. The ADL scale is based on seven levels of self-performance including dressing, eating, toilet use, bathing, mobility in bed, locomotion and transfer. Similarly, the IADL scale is based on seven levels of self-performance including meal preparation, housework, managing finance, phone use, shopping, transportation and managing medications Symptoms of depression were assessed through the Beck Depression Inventory (BDI) [19]. The BDI is a 21-item, patient-rated scale that has been validated in the hemodialysis population [20,21,22]. Scores can range from 0 to 63, with higher scores indicating more severe depression. In patients with end-stage renal disease, the BDI correlates highly with diagnostic criteria of depression, quality of life, functional status, severity of illness and mortality over time [20,21,22]. In a recent study of HD patients, the BDI was validated against a structured clinical interview for depression (SCID) suggesting a BDI score of ≥16 as an optimal cut-off for significant depression symptoms [22].

### 2.5. Statistical Analyses

Statistical analysis was performed by using the Statistical Package for Social Science (SPSS), release 15.0. Continuous variables were expressed as the mean ± SD and median [95% CI] or median [min–max], categorical variables displayed as frequencies (%) and the Mann–Whitney U test was used to assess significance of the differences between groups. A correlation matrix of PPI use, PDF and TIRD was built, reporting Spearman’s rho coefficient and significance values. After adjustment for multiple measures, a *p*-value < 0.01 was considered statistically significant.

## 3. Results

Three hundred and ninety-five patients were screened. Forty-nine were excluded according to exclusion criteria and three hundred and forty-six patients were included in this study. Of these, 259 used PPI and 87 did not. Their demographic, clinical, and laboratory characteristics are shown in Table 1. The two groups did not differ significantly for age, sex distribution, dialytic age, primary cause of ESRD, BMI, ADL, IADL, hemoglobin, serum albumin, serum creatinine, and dialysate sodium concentration. Interdialytic weight gain, Charlson comorbidity index score and dialysate temperature were significantly higher in PPI non-users (Table 1).

Two hundred and thirty-two patients (67%) declared PDF and 114 (33%) did not. Median [min–max] PDF frequency was 4 [0–5]. Median [min-max] PDF intensity was 3 [0–5]. Median [min–max] PDF duration was 3 [0–5]. Median [min-max] PDF sum was 9 [0–15]. The median [min–max] TIRD was 210 min [0–1440].

**Figure 1:**
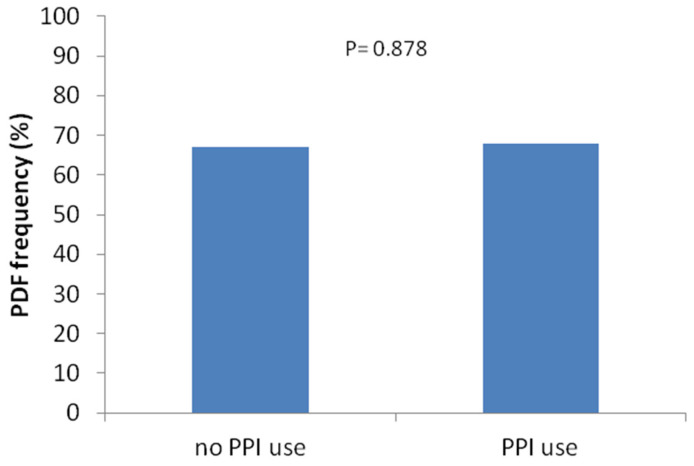
Frequency of PDF in PPI Users and PPI Non-Users.

**Figure 2:**
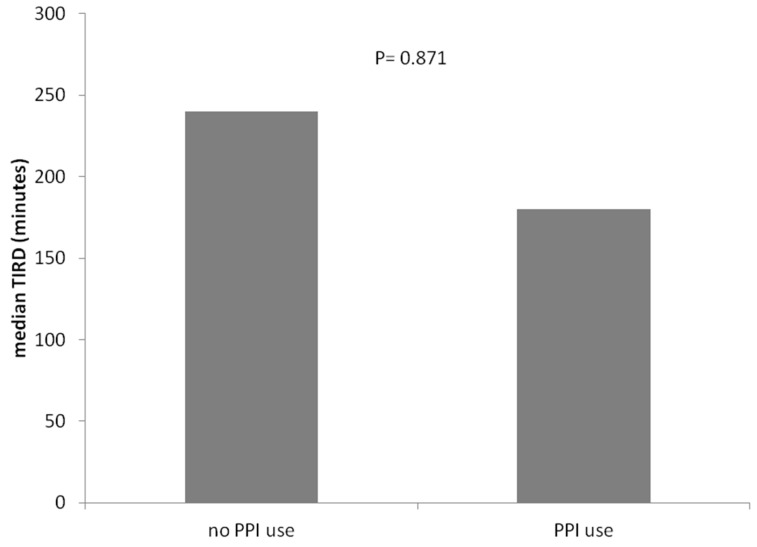
Length of TIRD in PPI Users and PPI Non-Users.

The prevalence of PDF was similar between PPI users and PPI non-users (Figure 1). Similarly, the length of TIRD (median [min–max]) did not differ significantly between PPI users (180 [0–1440] minutes) and PPI non-users (240 [0–1440]; *p* = 0.871) (Figure 2).

As shown in Table 2, the median PDF intensity, duration, frequency, and severity did not differ significantly between PPI users and PPI non-users.

Among the PPI users, 55 (21.2%) used omeprazole, 63 (24.3%) esomeprazole, 54 (20.8%) pantoprazole, 87 (33.6%) lansoprazole, and 7 (2.7%) rabeprazole. The prevalence of PDF was similar among the different types of PPI use and did not differ with respect to PPI non-users (Table 3).

Duration of PPI exposure was <1 year in 40 (15.4%) patients and ≥1 year in 219 (84.6%) patients. The prevalence of PDF did not differ significantly between the two exposures (Table 3).

As detailed in Table 4, the correlation matrix between PPI equivalent dose and PPI treatment duration and PDF frequency, PDF characteristics, TIRD, ADL, IADL and BDI showed whether there was statistical significance.

## 4. Discussion

Worldwide, the use of PPI is very common in patients on chronic hemodialysis for the prevention and treatment of gastroesophageal reflux disease or dyspepsia [13]. The use of PPI in chronic hemodialysis patients has been associated with many adverse events such as hip fracture, hypomagnesemia, abdominal aortic calcification, and all-cause mortality [23,24,25,26]. A recent Japanese study has also shown a significant association between the use of PPI and hyporesponsiveness to erythropoiesis-stimulating agents in hemodialysis patients [27].

More recently, the study of Knobbe et al. demonstrated that the use of PPI was independently associated with fatigue severity and lower quality of life among kidney transplant recipients, possibly as consequence of micronutrient malabsorption secondary to the lack of acidity in the stomach, disturbance of the gut–brain interaction, and, finally, of the inhibition of the vacuolar ATPase in many cells of the body with consequent detrimental effects in many physiological processes [12]. The association between PPI use and fatigue was present among all assessed PPI types and dose dependent [12].

Although these mechanisms may occur also in patients on chronic hemodialysis receiving PPI, in the present study we found that the use of PPI was not associated with PDF prevalence, PDF characteristics, and PDF severity in such patients. In addition, we demonstrated that the dose of PPI or the duration of the use of PPI was not independently associated with PDF and its characteristics as well as with ADL, IADL, and BDI. Interestingly, the present study also showed that no PPI was associated with PDF and PDF characteristics.

With respect to the study of Knobbe et al., beyond a different studied patient population, we determined the prevalence and characteristics of PDF and not fatigue of every day. PDF is a multifactorial event that develops or worsens after the hemodialytic session and may persist for hours. Its prevalence of PDF ranges from 20 to 86%, likely due to a variation in methods of ascertainment and participant characteristics. Although many variables have been found to be associated with PDF such as depression, sedentary behavior, functional disability, ultrafiltration volume, intradialytic hypotension, and branched chain amino acid depletion, the causes of PDF are not clearly understood [28]. Nevertheless, it is amenable that studies investigating the possible relationship between fatigue of non-dialysis days and the use of PPI will be run in the future. In fact, post-dialysis fatigue and fatigue of non-dialysis days seem to be two different entities with possibly different underlying mechanisms [28].

The present study also demonstrated that the use of PPI as well as the duration and dose of PPI were not associated with TIRD. This is the first time that this possible association has been investigated. TIRD has been shown to be associated with fatigue and other quality of life or stress scales, as well as social–leisure activity [15].

The present study has some limitations. First, this is an observational and crossover study, so no conclusion can be drawn about causality. Second, the sample size is relatively small.

In conclusion, the use of PPI in patients on maintenance hemodialysis is not associated with PDF and time of recovery after dialysis. The lack of association between the use of PPI and the prevalence and characteristics of PDF and the length of TIRD may suggest that PPI use in patients on maintenance hemodialysis is safe in terms of fatigue and recovery time.

## Figures and Tables

**Table 1:** Baseline demographical, clinical and laboratory characteristics. Data are shown as the mean ± standard deviation or median [95% CI for the median] for continuous variables. PDF: post-dialysis fatigue; BMI; body mass index; CCI: Charlson comorbidity index; ADL: activity of daily living; IADL, instrumental activities of daily living; TIRD, time of recovery after dialysis.

	PPI Non-Users (87)	PPI Users (259)	*p*
Age (years)	68 ± 14	68 ± 15	0.855
Sex: male/female	61%/39%	58%/42%	0.590
Dialysis age, months	61 ± 47 52 [6–240]	65 ± 57 54 [6–396]	0.836
Primary cause of ESRD (*n*)			
Hypertension	25	73	0.988
Diabetes	22	67	
Glomerulonephritis	7	27	
Polycystic renal disease	8	19	
Interstitial nephritis	7	17	
Other nephropathies	4	12	
Unknown	14	44	
BMI	25.2 ± 4.9 24.3 [17.8–47.3]	24.7 ± 4.7 23.6 [13.9–39.1]	0.263
CCI	2.2 ± 1.6 2 [0–7]	2.7 ± 1.7 3 [0–8]	0.025
ADL	5 ± 2 6 [0–6]	5 ± 1 6 [0–6]	0.841
IADL	6 ± 3 7 [0–8]	6 ± 3 8 [0–8]	0.370
TIRD	344 ± 361 240 [0–1440]	331 ± 305 180 [0–1440]	0.871
Weight gain (Kg)	1.4 ± 1.3 1.4 [0–4.5]	0.9 ± 1.2 3.0 [0–5.0]	0.002
Hemoglobin (g/dL)	11.3 ± 1.2 11.3 [8.1–14.1]	11.0 ± 1.2 11.2 [7.4–14.2]	0.132
Serum albumin (mg/dl)	36 ± 4 38 [29–41]	36 ± 6 38 [20–50]	0.772
Serum creatinine (mg/dL)	9.3 ± 2.8 9.2 [3.5–15.8]	9.0 ± 3.0 8.5 [1.9–18.9]	0.295
Dialysate sodium (mmol/L)	135 ± 13 139 [103–144]	134 ± 13 138 [103–145]	0.256
Dialysate temperature (°C)	36.6 ± 0.5 37 [36–37]	36.4 ± 0.5 36.5 [35–37]	0.025

**Table 2:** PDF intensity, duration, frequency and severity and TIRD according to the use of PPI. Data are expressed as the mean ± standard deviation and as median [min–max].

	All Patients (*n* = 346)	PPI Non-Users (*n* = 87)	PPI Users (*n* = 259)	*p*
PDF intensity	3.2 ± 1.3 3 [0–5]	3.3 ± 1.2 3 [0–5]	3.2 ± 1.4 3 [0–5]	0.598
PDF duration	3.3 ± 1.3 3 [0–5]	3.3 ± 1.3 3 [0–5]	3.3 ± 1.4 3 [0–5]	0.788
PDF frequency	3.5 ± 1.5 4 [0–5]	3.6 ± 1.3 4 [0–5]	3.4 ± 1.5 4 [0–5]	0.451
PDF severity	7.5 ± 5.3 9 [0–15]	8.0 ± 5.1 10 [0–15]	7.3 ± 5.4 9 [0–15]	0.402

**Table 3:** Type of PPI (%) and duration of PPI exposure (%) in patients with and without PDF.

	PDF Absent (*n* = 114)	PDF Present (*n* = 232)	*p*
Type of PPI			0.175
No use	25	22	
Omeprazole	13	17	
Esomeprazole	16	19	
Pantoprazole	12	17	
Lansoprazole	33	22	
Rabeprazole	1	3	
Duration of PPI exposure			0.115
None	26	22	
<1 year	7	15	
≥1 years	67	63	

**Table 4:** Correlation matrix between PPI equivalent dose, treatment duration and PDF frequency, PDF characteristics, TIRD, ADL, IADL and BDI [Spearman’s correlation coefficient and (statistical significance)].

	Equivalent Dose (mg)	Treatment Duration
PDF	0.05 (0.383)	−0.08 (0.220)
PDF Frequency	−0.08 (0.273)	−0.06 (0.455)
PDF Duration	−0.10 (0.158)	0 (0.967)
PDF Intensity	−0.08 (0.296)	0.02 (0.830)
PDF Severity	−0.01 (0.839)	−0.08 (0.230)
TIRD	−0.05 (0.466)	−0.01 (0.898)
ADL	0.04 (0.503)	−0.08 (0.199)
IADL	0.07 (0.256)	−0.11 (0.075)
BDI	0.08 (0.386)	0.09 (0.349)

## Data Availability

Data are contained within the article.
